# Peer support interventions for individuals with acquired brain injury, cerebral palsy, and spina bifida: a systematic review

**DOI:** 10.1186/s12913-019-4110-5

**Published:** 2019-05-08

**Authors:** Ben B. Levy, Dorothy Luong, Laure Perrier, Mark T. Bayley, Sarah E. P. Munce

**Affiliations:** 1 Toronto Rehabilitation Institute – University Centre, 550 University Avenue, Toronto, Ontario M5G 2A2 Canada; 2 Toronto Rehabilitation Institute – Rumsey Centre, 345 Rumsey Road, Toronto, Ontario M4G 1R7 Canada; 3 University of Toronto Libraries, 130 St. George Street, Toronto, Ontario M5S 1A5 Canada

**Keywords:** Peer support, Acquired brain injury, Cerebral palsy, Spina bifida, Community integration, Quality of life, Systematic review

## Abstract

**Background:**

Neurological disorders may negatively impact community integration and/or quality of life. Peer support has emerged as a potential strategy to enhance patients’ efficacy in managing their own health. This review examines the key characteristics and impact of peer support interventions for adults with acquired brain injury, cerebral palsy, and spina bifida on community integration and quality of life.

**Methods:**

Eligible studies reported on peer support interventions for adults (16 years of age or older) with acquired brain injury, cerebral palsy, or spina bifida. Only randomized controlled trials published in English in the last 10 years were included. MEDLINE, EMBASE, PsycINFO, and CINAHL were used to conduct the literature search. Two reviewers independently screened studies, abstracted data, and evaluated the risk of bias (for individual study elements and overall) using the Cochrane Risk of Bias Tool.

**Results:**

The systematic review included 6 trials reporting on acquired brain injury only. Of these studies, 4 reported on stroke and 2 reported on traumatic brain injury. Two studies found significant improvements in quality of life following peer support. No studies reported significant results on community integration. Considerable heterogeneity existed in the key characteristics of interventions.

**Conclusions:**

There are a limited number of studies on the impact of peer support interventions for adults with acquired brain injury, cerebral palsy, or spina bifida on community integration and quality of life. Standardization of key intervention characteristics may aid the global adoption of peer support as a formalized, evidence-based practice.

**Electronic supplementary material:**

The online version of this article (10.1186/s12913-019-4110-5) contains supplementary material, which is available to authorized users.

## Background

Globally, neurological disorders are the leading cause group of disability-adjusted life years and the second-leading cause group of death [1]. As the burden of neurological diseases and resulting demand for health services continue to increase worldwide, global shortages of rehabilitation professionals have urged further innovation within existing health systems and more efficient resource allocation [1]. The development and adoption of community-based programs, particularly non-traditional models of care such as self-management, have stemmed from the existing burden of neurological disorders and the need for further efficiencies in treatment [2].

Neurological conditions including acquired brain injury (ABI), cerebral palsy (CP), and spina bifida (SB) frequently have significant social and psychological implications that may negatively impact an affected individual’s community integration (CI) and/or quality of life (QoL) [3–5]. These conditions can impede patients’ abilities to participate in meaningful activities, such as employment, and raise numerous challenges including isolation and disability-associated stigma [6–8]. Gaps in the provision of health care services, including their limited availability for rare neurological disorders (such as cerebral palsy and spina bifida), restrictions on patients presenting with comorbidities (e.g., mental illness), and imbalanced allocation of resources, may further restrict the community integration and/or QoL of patients and their caregivers [6]. In addition to treatments administered by health care professionals (e.g., rehabilitation therapy), individuals with these neurological conditions may rely extensively on informal caregivers such as family members, friends, or neighbours for general assistance, transportation, and regular emotional support [6]. Caregivers provide unpaid support and thus carry an increased burden of care (i.e., physical, mental, and financial) over the next decades [9]. For these reasons, there is an imperative for individuals with neurological conditions to self-manage their condition(s) if they possess the capacity (i.e., energy, information, and time) and responsibility (i.e., understanding of their specific role in carrying out self-management tasks) required to do so [10]. In their recent analysis on the future of health and social care services for Canadian seniors, the Conference Board of Canada suggested that the onus is on governments and key stakeholders to begin examining creative approaches to sustainably enhance the availability of supports for patients in need [9]. Peer support has emerged as a promising alternate intervention for use alongside other treatments; an effective and cost-effective self-management method to counter many of the limitations (i.e., gaps in the provision of health care) present in the current health care context [11].

Peer support is defined as support for a person with a chronic condition from someone with the same condition or similar circumstances [11, 12]. Its defining attributes are emotional, informational, and appraisal support; these 3 attributes are ideally used in combination to attain 1 or more given health outcomes including improvements in mental or physical health [13]. Emotional support aids in enhancing or restoring self-esteem through caring and encouragement; informational support provides pertinent advice, facts, or suggestions to resolve problems; and appraisal support motivates individuals to overcome stressors by affirming their emotions, thoughts, and actions [13]. Interventions that include these forms of support, rooted in experiential knowledge, can be delivered in a variety of different protocols (i.e., frequency, duration, and setting for session delivery) [13]. There is emerging evidence of effectiveness for some of these protocols in improving community integration and QoL [14, 15]. Current variability in the structuring and implementation of peer support however, suggests that its global adoption as a formalized, evidence-based practice may first require the development of a more standardized approach to key intervention aspects, particularly the training and certification of mentors [16].

Thus, there is a need to synthesize the evidence on the impact of peer support interventions (PSIs) on community integration and QoL for individuals affected by acquired brain injury, cerebral palsy, and spina bifida. The populations included in the current review are consistent with the populations selected in Turk et al.’s review on adults with childhood onset disabilities, which included cerebral palsy, spina bifida, and childhood onset cancer, particularly pediatric brain neoplasm [17]. Given that acquired brain injury can occur at any age, this review has broadened the time of onset of acquired brain injury to childhood or adulthood. Previous studies have reported on the efficacy of peer support interventions for a range of conditions including asthma [18], autism spectrum disorder [19], cancer [20], depression [21], and diabetes [22], yet the literature to date has not examined the key characteristics of peer support interventions as related to community integration and QoL outcomes in acquired brain injury, cerebral palsy, and spina bifida. Therefore, the specific research objectives of this systematic review are: (1) to determine the impact of peer support interventions for adults with acquired brain injury, cerebral palsy, and spina bifida on community integration and QoL; and, (2) to identify the key characteristics of peer support interventions for adults with acquired brain injury, cerebral palsy, and spina bifida. The results of the current review are expected to inform the enhancement of existing peer support programs and guide the development of future peer support interventions to best serve the needs of all involved in the rehabilitation process. Gaining insight into the key characteristics of these interventions may ultimately lead to improvements in their implementation and efficacy.

## Methods

This systematic review was registered with PROSPERO (CRD42018102386) and drafted in accordance with the Preferred Reporting Items for Systematic Reviews and Meta-analyses (PRISMA) statement [23].

### Eligibility criteria

To be eligible for inclusion, studies must have investigated peer support interventions for adults (16 years of age or older) with acquired brain injury, cerebral palsy, or spina bifida. Peer support is defined as support for a person with a chronic condition from someone with the same condition or similar circumstances [11, 12]. This type of support can be delivered through several formats, including face-to-face meetings, telephone calls, and online interventions (e.g., peer-to-peer video conferencing, social media peer interactions, peer-to-peer text messages). Studies reporting on all types of peer support interventions were included, irrespective of characteristics such as their duration or frequency.

Outcomes of interest included measures of community integration – defined by employment or other productive activity, independent living, and social activity [24] – or QoL – defined as an individual’s perception of their position in life in the context of the culture and value systems in which they live and in relation to their goals, expectations, standards, and concerns [25]. Included studies measured community integration and/or QoL using validated scales, classifications, and/or measurement systems. To ensure the inclusion of all literature relevant to the current health care context, this review was limited to studies published in the last 10 years (i.e., from January 2008 to June 2018). It included randomized controlled trials only. Studies involving comorbidities (including mental illness) were accepted. Only English-language publications were included. Conference abstracts and proceedings were excluded.

### Search strategy and information sources

Literature search strategies were developed by an experienced informational specialist (LP) using medical subject headings and text words related to the conditions of interest (i.e., ABI, CP, or SB) and peer support interventions. The search was peer-reviewed by an experienced librarian using the Peer Review of Electronic Search Strategies (PRESS) checklist and modified as necessary [26]. MEDLINE, EMBASE, PsycINFO, and CINAHL were searched on June 8, 2018. Appropriate wildcards were used in the searching to account for plurals and variations in spelling. The search strategy for MEDLINE can be found in an additional file [see Additional file 1]. Reference lists from reviews and selected articles were hand searched to ensure literature saturation.

### Study selection and data abstraction

Titles and abstracts identified by the literature search were screened (i.e., level 1 screening). To determine final inclusion, full text screening of potentially relevant articles (i.e., level 2 screening) was completed. Screening at both level 1 and level 2 was done independently by 2 reviewers (BBL and DL). The *κ* statistic was calculated for articles in level 1 screening and articles eligible for level 2 screening to measure inter-rater reliability [27]. Conflicts between reviewers were resolved by an additional reviewer experienced in the area of research, or by discussions to reach consensus.

Data abstracted included the authors, year of publication, country of study, recruitment setting, mean age and age range, sample size, sex, type and severity of condition, description of the intervention, key characteristics of the intervention (operationalized as delivery setting, session duration, frequency, program length, administrator(s) of the intervention, training or certification of administrator(s), underlying theories or theoretical frameworks, and type of support provided), and intervention-related outcomes.

### Risk of bias assessment

Two reviewers (BBL and DL) independently assessed the risk of bias (i.e., as low, high, or unclear) in the included studies for individual elements found within the Cochrane Risk of Bias Tool for randomized controlled trials and assigned an overall risk of bias rating [28].

## Results

### Study selection

The literature search yielded 5713 records. MEDLINE, EMBASE, PsycINFO, and CINAHL retrieved 1423, 1947, 1303, and 1040 records, respectively. Hand searching yielded 1 additional study for a total of 5714 identified records. 4797 records remained following the removal of duplicates. In level 2 screening, 20 full-text articles were assessed and 14 were excluded. Reasons for exclusion included wrong study type (i.e., not a randomized controlled trial), wrong intervention (i.e., not peer support), wrong population (i.e., not ABI, CP, or SB), wrong outcome(s) (i.e., not CI or QoL), or wrong publication type (i.e., conference abstract). One conference abstract was excluded as it accompanied 2 full-text studies. Several attempts were made to contact the authors of the 3 additional conference abstracts; however, full-text articles could not be obtained.

**Fig. 1:**
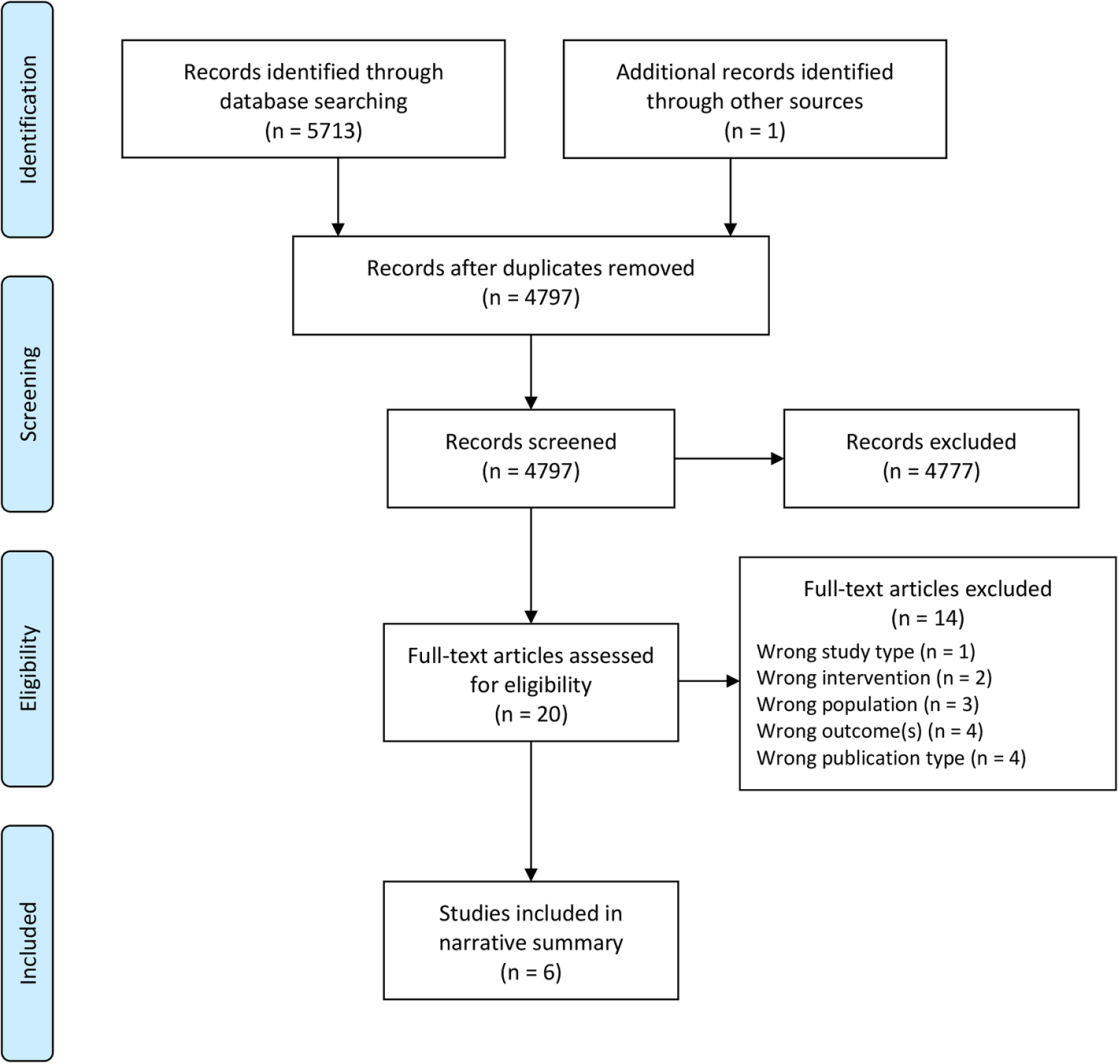
Identified studies from the literature search and screening process. Modified from the Preferred Reporting Items for Systematic Reviews and Meta-analyses (PRISMA) statement [23]

Six studies remained for final inclusion in the narrative summary. It was not possible to conduct a meta-analysis given the low number of articles that made it to extraction and the heterogeneity of outcome measures. Therefore, the studies were synthesized descriptively with a focus on study characteristics, key intervention characteristics, and outcomes. The *κ* statistic was found to be 0.80 for level 1 screening and 1.00 for level 2 screening, indicating strong and almost perfect agreement for each stage, respectively [27]. Figure 1 outlines the systematic review process using a PRISMA flow diagram.

### Identified studies

Included studies were conducted in Australia, the Netherlands, the United Kingdom, and the United States, and published between 2011 and 2015. Participant recruitment settings included rehabilitation centres and (rehabilitation) hospitals. Study sample sizes ranged from 30 to 153 participants and mean ages ranged from 31.7 to 69.4 years. Percentages of female participants ranged from 11 to 59%. Only 1 study had a majority of females [29]; the second-highest percentage of females was 45.1%. Out of 6 included studies, 4 reported on stroke [29–32] and 2 reported on TBI [33, 34]. No studies reported on cerebral palsy or spina bifida. The main characteristics of each included study can be found in Table 1.

**Table 1:** Summary of characteristics of included studies (*n = 6)*

Author	Year	Country	Recruitment Setting	Mean age (SD); Age range	Sample size	Females (%)	Condition
Aben et al. [30]	2013	Netherlands	Multiple rehabilitation centres	58 (9.7); Not specified	153	45.1	Stroke
Aben et al.^a^ [31]	2014	Netherlands	Multiple rehabilitation centres	58 (9.7); Not specified	153	45.1	Stroke
Cadilhac et al. [29]	2011	Australia	Multiple hospitals	69.4 (11.4); 62–78	143	59	Stroke
Hanks et al.^b^ [33]	2012	United States	Rehabilitation hospital	Intervention: 38.46 (17.60), Control: 40.90 (17.33); 17–86	96	11	TBI
Stamatakis [32]	2015	United Kingdom	Stroke rehabilitation centre	65.8 (12.8); 40–89	47	44.7	Stroke
Struchen et al. [34]	2011	United States	Multiple rehabilitation hospitals	31.7 (11.7); 21–68	30	20.0	TBI

*SD* standard deviation, *TBI* traumatic brain injury

^a^Extension of Aben et al.’s 2013 study to include a 1-year follow-up period

^b^Data for TBI group only

### Intervention characteristics

The specific descriptions of the peer support interventions used in each of the included studies differed considerably. Interventions could generally be classified into 2 categories based on the interactions that occurred: group-to-peer interventions had multiple participants assigned to 1 or more peers; and individual-to-peer interventions had single participants each assigned to a single peer (though in this case, the same peer could be assigned to multiple participants).

Group interventions involved scheduled peer support sessions. During these sessions, participants shared problems they experienced in their daily lives, received education on disability-specific topics such as recovery, and contributed to discussions on participant-identified topics including psychosocial difficulties. Group interventions all occurred in person and delivery settings included rehabilitation centres and hospitals.

Individual interventions focused on disability-specific education, social and emotional support, and improving social integration. In contrast to group interventions, participants in individual interventions had the option of using phone calls, emails, and/or in-person meetings to communicate with their assigned peer; however, specific guidelines for usage differed between studies. For example, Hanks et al.’s study [33] permitted the form(s) of communication to be decided based on the mentee’s preferences, though the other individual-to-peer study by Struchen et al. [34] regarded phone calls and emails as supplements to compulsory in-person meetings. Both individual interventions occurred in the community.

For both categories of interventions, session durations ranged from under 15 min to 2.5 h, though group interventions all had a minimum session duration of 1 h. In Hanks et al.’s study [33], the majority (85%) of sessions lasted less than 1 h, and two thirds (67%) of sessions lasted under 30 min. Struchen et al.’s study [34] did not specify session durations. Sessions occurred either weekly, twice per week, monthly, or twice per month for all included studies. One intervention; however, changed the frequency of contact 3 times throughout the study period as participants progressed through the program (i.e., guidelines specified once per week contact for the first month, twice per week contact for the next 2–3 months, and once per month contact for the remainder of the intervention) [33]. The majority of interventions lasted 1 to 3 months and the longest lasted for 1 year [33].

Peers served as administrators of the peer support intervention in 4 of the 6 included studies [29, 32–34]. In 2 of these cases, health educators or clinicians co-facilitated sessions alongside peers [29, 32]. Though peers generally served comparable roles, each of the 4 studies using peers as administrators referred to them by slightly different titles: peer leaders, peer mentors, peer supporters, and social peer mentors. The 2 remaining studies used psychologists to conduct their peer support interventions and derived their peer support element from the interactions that occurred between group members [30, 31].

Methods of training or certifying administrators for their roles differed between the studies. Four interventions included training [29, 32–34], though only 3 studies reported on the methodology of the training component [32–34]. In the studies that described the training component, role-specific training generally included skill building across multiple domains (e.g., communication, group facilitation, and mentorship), role-playing, and discussions on disability and peer support. The 3 studies that specified the durations of their training programs reported total lengths of 2, 3, and 20 h [32–34]. Training facilitators/administrators differed in their disciplines and included a program manager for a stroke foundation, a study author and supervisor, clinicians, a neuropsychologist, and a consumer representative with TBI. The 2 studies that used a psychologist to run their peer support interventions did not specify if he or she received any form of additional training prior to administrating the intervention [30, 31].

No underlying theories or theoretical frameworks were specified for the peer support interventions in 5 of the 6 included studies. The remaining intervention by Hanks et al. [33] used a supported-employment framework where mentors were hired as contingent employees and provided with supervision by professionals on a weekly basis. Though self-efficacy (i.e., the confidence an individual has in their ability to affect their own health or perform self-management behaviours) was referenced as being important to the development of Cadilhac et al.’s generic intervention [29] (i.e., an arm of the study that did not include peer support), it was unclear if self-efficacy was incorporated into their peer support intervention, and no related measures of social cognitive theory (e.g., mastery) were used [35].

Three peer support interventions made use of all 3 forms of support (i.e., emotional, informational, and appraisal) [29, 32, 34]. One peer support intervention provided both informational and emotional support [33], and the 2 remaining peer support interventions used informational support as their sole type of support [30, 31]. The majority of studies did not mention any of the 3 attributes directly (i.e., by using the terms emotional support, informational support, or appraisal support) in descriptions of their peer support intervention. The key characteristics of each peer support intervention can be found in Table 2. Moreover, a summary of each peer support intervention using the Template of Intervention Description and Replication (TIDieR) framework can be found in an additional file [see Additional file 2].

**Table 2:** Summary of key characteristics of peer support interventions in included studies (*n = 6)*

Author; Year	Description of peer support intervention	Delivery setting	Session duration; Frequency; Program length	Administrator(s); Training or certification of administrator(s)	Underlying theories or theoretical frameworks	Type of support provided (i.e., emotional, informational, and/or appraisal)	Main findings on CI and/or QoL
Aben et al.; 2013 [30]	Peer support group in which general education on causes and consequences of stroke was provided; patients were invited to share problems experienced in their daily lives	Rehabilitation centre; Group	1 h; Twice per week; 4.5 weeks	Psychologist;Not specified	Not specified	Informational	No significant results for QoL utility score (*p* = 0.459), QoL VAS (*p* = 0.307), psychological QoL (*p* = 0.089), or social-related QoL (*p* = 0.200)
Aben et al.; 2014^a^ [31]	Peer support group in which general education on causes and consequences of stroke was provided; patients were invited to share problems experienced in their daily lives	Rehabilitation centre; Group	1 h; Twice per week; 4.5 weeks	Psychologist; Not specified	Not specified	Informational	Psychological QoL improved significantly (*p* = 0.030) for patients under 65 years old in a Memory Self-efficacy training program compared to the PSI; no significant results for QoL VAS (*p* = 0.549), social-related QoL (*p* = 0.174), or psychological QoL for all participants (*p* = 0.077)
Cadilhac et al.; 2011 [29]	Stroke-specific Self-Management Program; differs from the self-management program developed by Stanford University since it only includes stroke survivors, has greater contact time, is only delivered by health professionals and peer leaders skilled in stroke and trained by the National Stroke Foundation, provides targeted stroke-specific information each week, and revisits information provided in other weeks to ensure retention of learning and skills	Hospital; Group	2.5 h; Once per week; 8 weeks	Peer leaders and health professionals (i.e., stroke educators); Training program run by the National Stroke Foundation Program Manager	Not specified	Emotional, informational, appraisal	No significant differences observed between groups; large effect sizes
Hanks et al.; 2012 [33]	Mentoring sessions with discussions focusing on emotional well-being, post-TBI QoL, and CI; mentors helped mentees gain access to community resources and discussed TBI- or caregiving-related topics through phone calls, emails, or in-person meetings	Community; Individual	Ranged from 5 min to more than 1 h; Minimum of once per week for the first month, twice per week for the next 2–3 months, and once per month for the remainder of the year; 1 year	Peer mentors; Mentors participated in 20 h of training, including modelling interviewing skills with a supervisor and fellow trainees, telephone role playing, discussion of what is and what is not mentoring, and communication skills and active listening; mentors were also evaluated on social competency, willingness to talk openly about disability and life experiences, motivation, and commitment to participation by training staff	Supported-employment model; mentors were hired as contingent employees and involved in weekly in-person supervision from a psychologist, nurse, and community outreach coordinator	Informational, emotional	Health-related QoL improved significantly (*p* = 0.04) for TBI patients in the PSI, compared to the non-mentored control group; no significant results for CI (*p* = 0.35)
Stamatakis; 2015 [32]	Peer support sessions during which participants identified topics related to post-stroke rehabilitation that they wanted to focus on; topics identified were common psychosocial difficulties and practical considerations and were often discussed in more than 1 session	Day hospital; Group	1.5–2 h; Once per week; 5 weeks	Peer supporters and clinicians; Peer supporters attended a 3-h training session facilitated by the author and study supervisor alongside clinicians in the local service, consisting of information about the proposed group, theoretical knowledge/rationale (about stroke and peer support as a model), and group facilitation skills, involving a combination of teaching, working in pairs, observation, and role-play	Not specified	Emotional, informational, and appraisal	QoL improved significantly (*p* = 0.003) for patients in the PSI; no significant results for CI (*p* > 0.05)
Struchen et al.; 2011 [34]	Social peer mentoring program for improving social integration; goal of outings was to foster increased social networking for the peer partner through introductions to people, activities, and resources within the peer partner’s own community; phone calls and emails could be used to supplement required in-person meetings	Community; Individual	Not specified; Twice per month (minimum; not always met); 3 months	Social peer mentors; Initial training was led by a neuropsychologist and a consumer representative with TBI who was part of the research team; conducted in 2-h group sessions including didactic presentation, discussion, and role-play of specific skill-building activities to ensure an understanding of the mentor role, introduce and practice specific content for the facilitation of skill building by the peer partner, review safety issues and how to handle crisis situations, and understand documentation responsibilities	Not specified	Emotional, informational, and appraisal	No significant results on CI; mean community participation scores increased from 68.5 to 79.8 after the PSI; scores on the majority (83%) of Social Activity Interview items increased after the PSI, including *Satisfaction with social life for past month* (*p* = 0.08); scores on all Weekly Social Activity Survey items increased after the PSI

*CI* community integration, *PSI* peer support intervention, *QoL* quality of life, *TBI* traumatic brain injury, *VAS* visual analogue scale

^a^Extension of Aben et al.’s 2013 study to include a 1-year follow-up period

### Impact of peer support interventions on community integration and quality of life outcomes

Community integration and QoL outcome data were reported in 4 [29, 32–34] and 5 [29–33] studies, respectively. Community integration was measured using the Health Education Impact Questionnaire (heiQ) [36], Community Integration Measure (CIM) [37], Barthel Index (BI) [38], Craig Handicap Evaluation and Reporting Technique (CHART) [39], Social Activity Interview (SAI) [34], and Weekly Social Activity Survey (WSAS) [34]. QoL was measured using the EQ-5D [40], WHOQoL-BREF [41], Assessment of Quality of Life (AQoL) [42], and Medical Outcomes Study 12-Item Short Form Health Survey (SF-12) [43].

Improvements in community integration and/or QoL were noted in 5 out of 6 studies [29, 31–34]; however, only 2 studies found statistically significant improvements following participants’ completion of a peer support intervention [32, 33]. Furthermore, statistically significant results related to QoL only, though 1 study did report a positive trend for community integration [34]. In the study by Hanks et al. [33], health-related QoL, as measured by the physical functioning scale of the SF-12, increased significantly (*p* = 0.04) after 1 year for TBI patients following the peer support intervention. The study by Stamatakis [32] reported a significant improvement in QoL (*p* = 0.003), as measured by the EQ-5D-3 L, after 9 weeks for peer support intervention patients. The mean EQ-5D-3 L score of peer support intervention participants in this study dropped from 9.7 to 9.0, in contrast to the mean score of participants in the control group, which dropped from 10.0 to 9.9 (lower scores are associated with increased QoL). No other studies reported statistically significant data on the impact of peer support interventions on either community integration or QoL.

Struchen et al. [34] determined that community integration, as measured by the *Satisfaction with social life for the past month* item in the SAI, increased by a greater extent after 3 months for patients who participated in the peer support intervention than for those in the wait-list control group; however, this result was reported as a trend only (*p* = 0.08). Additionally, Aben et al.’s 2014 study [31] found a significant improvement in psychological QoL (*p* = 0.030), as measured by the psychological QoL domain on the WHOQoL-BREF, after 1 year for patients 65 years old or under, though this result applied to participants in the study’s Memory Self-efficacy training program, rather than the peer support intervention. The main findings on community integration and/or QoL in each study are summarized in Table 2.

### Risk of bias assessment

Four studies received a low overall risk of bias rating [29–32] and 2 studies received an unclear overall risk of bias rating [33, 34]. Sequence generation was judged as high risk for 1 study [33] and unclear risk for another study [34]. Blinding of participants and personnel was rated as unclear risk for 4 studies [29, 32–34]. Blinding of outcome assessors and allocation concealment were also rated as unclear risk for 1 [34] and 2 [33, 34] studies, respectively. One study was judged as having an unclear risk for other sources of bias since it did not describe if and/or how baseline imbalances were accounted for [29]. Other studies appropriately accounted for any imbalances and received a low risk rating for this domain. Incomplete outcome data and selective reporting were rated as low risk for all studies.

Two thirds (67%) of included studies had a minimum of 1 domain judged to have unclear risk of bias [29, 32–34]. Blinding of participants and personnel was most commonly rated as unclear risk since many studies failed to provide detailed information on blinding procedures (particularly for participants) and performance bias due to knowledge of allocation. These findings limit the results of some of the included studies, including the findings of 1 study that reported significant QoL improvements but was rated as high risk for sequence generation and unclear risk overall [33]. In addition to uncertainties in several bias-related domains, included studies had a number of limitations. Several studies had large dropout rates/losses to follow-up; in 1 study, the twice-per-month minimum for in-person meetings was met only half of the time [34].

## Discussion

The purpose of this systematic review was to: (1) determine the impact of peer support interventions for adults with acquired brain injury, cerebral palsy and spina bifida on community integration and QoL; and, (2) identify the key characteristics of these interventions for this population. To the best of our knowledge, this is the first systematic review on peer support interventions to include cerebral palsy and spina bifida populations, as well as operationalize and synthesize the evidence on the key characteristics of peer support interventions. Studies generally reported improvements in community integration and/or QoL for peer support intervention patients. However, only 2 of the 6 included studies found statistically significant improvements in at least 1 outcome measure following participants’ completion of a peer support intervention, and these results related solely to QoL [32, 33]. Struchen et al. [34] reported a trend in community integration improvement for peer support intervention participants, though this result did not reach the threshold for significance. No studies on cerebral palsy or spina bifida were included in this review, revealing a need for future investigation on the impacts of peer support interventions on these populations. This is understandable given the shortened life expectancies of individuals with cerebral palsy and spina bifida, in addition to the focus in the literature on children with these conditions, rather than adults [17]. Furthermore, included studies were limited to stroke and TBI populations. Additional research on acquired brain injury populations of other origins (e.g., encephalitis, brain tumour) is justified.

Findings revealed a considerable heterogeneity in the key characteristics of peer support interventions across all studies. The absence of any underlying theories or theoretical frameworks supporting the majority of peer support interventions was notable, with only one study mentioning a model that informed the intervention (but which was not a theoretical framework) [33]. Studies generally reported that experts from relevant disciplines and/or organizations collaborated to develop the peer support interventions yet did not specify the method by which key characteristics such as session duration, frequency and program length were chosen, or how these types of decisions were informed. The integration of 1 or more empirical frameworks into peer support interventions may aid in the creation of more standardized intervention protocols that could serve to improve the implementation and efficacy of these interventions on a global scale. One model that may be suitable for the development of future peer support interventions may be the relationship perspective to social support, which posits that the health effects resulting from social support are tied directly to the relationship processes that occur alongside it (e.g., companionship, intimacy) [44]. Interventions designed to build meaningful relationships and comfortably integrate participants into more social settings over time could conceivably yield higher efficacy due to their capacity to improve self-esteem [44]. Social cognitive theory, pioneered by Bandura, may also serve as a suitable theoretical basis for interventions, particularly in relation to self-efficacy [45]. Tailoring interventions to encompass mastery of skills owing to sustained effort, observation of successful peers, and continuous encouragement and reinforcement from peers could enhance self-efficacy and benefit community integration and quality of life as a result [45–47]. Previous literature suggests that a systematic approach to intervention development, including a strong rationale for design and clear reporting of the intervention development process, is necessary [48], and some research suggests that a theory-informed intervention can lead to better outcomes. Thus, the absence of theory-informed interventions (with proposed mechanisms and impacts) in the included studies may explain the lack of statistically significant results.

The current review found that peer support interventions included a variety of support and combinations of support (i.e., emotional, informational, and appraisal) [13]. It is possible that the type(s) of support offered in a peer support intervention is/are associated with the effectiveness of the peer support intervention on community integration and/or QoL; however, further research is required to corroborate this theory. Both studies that reported a significant improvement in QoL after participation in a peer support intervention used a minimum of 2 of the 3 types of support. Moreover, both Cadilhac et al. [29] and Struchen et al. [34] used all 3 types of support and found large effect sizes and a trend in community integration improvement, respectively. Thus, it is also possible that peer support interventions that incorporate all 3 types of support may be more effective, but again, future studies and reviews should confirm this in other study populations. In addition, future studies could examine the nature of the relationship not only between peer support interventions and community integration and QoL, but also between these outcomes and efficacy to self-manage health.

Several studies catered to the specific needs of participants by permitting participants to choose their preferred mode of contact [33], identify relevant topics for discussion [32], or contact peers more frequently than intervention guidelines specified [34]. This approach typically reduced or eliminated logistical barriers (e.g., geographical distance) and may have improved outcomes. For example, both studies that reported significant improvement in QoL used at least 1 of the 3 aforementioned accommodations in their peer support interventions. Still, facilitators to patient participation in such programs, including sharing a background and/or sense of identity with one’s mentor and receiving support from health care staff, continue to be countered by barriers such as logistical challenges (e.g., difficulty scheduling mutually suitable meeting locations and/or times) [49]. Muller et al. have suggested integrating several forms of technology (e.g., smartphones, tablets) into peer support programs to minimize barriers including geographical distance and limited time (i.e., due to employment or other preoccupations) for both mentees and mentors [14, 49]. Online interventions (e.g., social media peer interactions) have also evolved to connect a larger and more diverse demographic, allowing a growing number of peers to relate personal experiences, seek explicit information, or offer explicit advice to others from the comfort of their personalized care setting [50]. Opportunities for participants to tailor their interventions to an appropriate degree, so that their personal preferences and/or goals can be met, should be investigated further as intervention protocols continue to evolve. Conceivably, implementing a standardized intervention protocol and simultaneously tailoring to personal preferences could be achieved by setting core components of the intervention that must be implemented, but also permitting some ability to tailor the intervention at the discretion of administrators.

The results of this review share similarities to another systematic review on the evidence for the use of peer support in the rehabilitation of acquired brain injury [51]. Conducted by Wobma et al. in 2016, the review included 2 studies (on TBI); both studies were also included in the current review. Similarly, Wobma et al. noticed great heterogeneity in intervention protocols and recommended that the characteristics of optimal dosage, length of peer support, and means of communication (e.g., in-person meetings compared to phone calls) be further investigated. Notably, a key difference between Wobma et al.’s study and ours is that Wobma et al. excluded acute survivors of acquired brain injury from the definition of peer supporters and only included rehabilitation-oriented studies (i.e., studies on topics such as secondary stroke prevention were excluded). Thus, no stroke studies qualified for inclusion and the authors ultimately concluded that the evidence for peer support is limited. The authors further acknowledged the importance of gaining more insight into the effects of peer support (i.e., on a range of health- and behaviour-related outcomes), in addition to intervention protocols. The current review implemented these suggestions by specifically examining community integration and QoL outcomes, as well as operationalizing and investigating the key characteristics of peer support interventions. Our review did not investigate phase of rehabilitation, selection of peers, or participant-to-peer match strength as key intervention characteristics as this information was not consistently available across all of the included studies; however, Wobma et al. suggested that these may be beneficial to examine in the future.

This systematic review is aided by several strengths; namely, the use of an experienced informational specialist to conduct an exhaustive literature search and the application of independent screening, data extraction and quality appraisal conducted by 2 reviewers in duplicate. The broad scope of the peer support definition used in this study is also of benefit given the considerable heterogeneity found in the key characteristics of interventions. This study also acknowledges some limitations. The literature search was limited to the last 10 years (i.e., from January 2008 to June 2018) and potentially excluded a number of relevant studies that may have met all other criteria for inclusion. Since only randomized controlled trials were included, relevant outcome data from other study types may have also been missed. If a broader range of study designs had been included, it is possible that other studies on peer support interventions for cerebral palsy and spina bifida populations may have been found. Furthermore, there may have been bias toward English-speaking countries since non-English studies were excluded and publication bias may have been introduced as a result of the decision to exclude conference abstracts [52]. Overall, the consistent use of the CONSORT guidelines for the reporting of randomized controlled trials would facilitate quality appraisal/risk of bias assessment and increase the transparency of the methods/results of trials included for a systematic review [53].

## Conclusions

This systematic review on peer support interventions for individuals with acquired brain injury, cerebral palsy and spina bifida revealed an overall paucity of studies on the impact of peer support on community integration and QoL outcomes for adults. The absence of studies on cerebral palsy and spina bifida, in addition to the considerable heterogeneity found in key intervention characteristics, justify the need for future research in this area of study. Using empirical theories to inform practice, increasing the types of support provided, and involving participants in the development of suitable interventions may ultimately enhance the implementation and efficacy of peer support interventions. Moreover, standardization may serve to advance the global adoption of peer support as a formalized, evidence-based practice.

## Additional files

Additional file 1: MEDLINE Search Strategy. (DOCX 29 kb)

Additional file 2: **Table S1.** Summary of peer support interventions *(n = 6)* using the TIDieR framework. [1] (DOC 46 kb)

## Abbreviations

ABI: Acquired brain injury
CI: Community integration
CP: Cerebral palsy
PSI: Peer support intervention
QoL: Quality of life
SB: Spina bifida
TBI: Traumatic brain injury
