# Supplementary material for: Peer support interventions for individuals with acquired brain injury, cerebral palsy, and spina bifida: a systematic review
Source: BMC Health Serv Res. 2019 May 8;19:288. doi: 10.1186/s12913-019-4110-5 (PMC6505073; doi:10.1186/s12913-019-4110-5)
Supplement: Supplementary file 1 — MEDLINE Search Strategy. (DOCX 29 kb) [file 12913_2019_4110_MOESM1_ESM.docx]

Table S1. Summary of peer support interventions *(n=6)* using the TIDieR framework. [1]

| ^Study^  _Item_ | Aben et al., 2013 [2] | Aben et al., 2014* [3] | Cadilhac et al., 2011 [4] | Hanks et al., 2012 [5] | Stamatakis, 2015 [6] | Struchen et al., 2011 [7] |
| --- | --- | --- | --- | --- | --- | --- |
| Brief name | Peer support group | Peer support group | Stroke-specific Self-Management Program (SSMP) | Peer-mentoring program for persons with TBI | Stroke peer support group | Social peer mentoring program for persons with TBI |
| Why | Used as a control intervention | Used as a control intervention | Disease-specific self-management programs may have advantages over generic programs; the SSMP is considered a viable alternative to the generic self-management program developed by Stanford University since it only includes stroke survivors, has greater contact time, and is only delivered by health professionals and peer leaders skilled in stroke | Mentored individuals were expected to demonstrate better emotional well-being and higher levels of community integration; used a supported-employment model where mentors were hired as contingent employees and were involved in weekly in-person supervision from a psychologist, nurse, and community outreach coordinator to maximize engagement | Peer support program may be effective in a community stroke service; hypothesized that participants attending a peer support program will report significantly reduced psychological stress and significant improvements in quality of life, perceived social support, and activities of daily living, mediated by the therapeutic value of the group | Social mentoring program for improving social integration in adults with TBI may be feasible and yield increased social functioning outcomes; mentors with TBI could serve as more meaningful role models since having themselves overcome barriers to social integration, they may be more understanding of the challenges faced by peer partners |
| What (materials) | The intervention group received a booklet with handouts of the peer support sessions after completing all sessions | The intervention group received a booklet with handouts of the peer support sessions after completing all sessions | Not specified | The curriculum and training manuals used were based on materials from local and national programs, and included introduction to the peer mentoring project, effects of traumatic brain injury and disability awareness, practical skills beneficial in any helping relationship (i.e., building trust, listening, responding, sharing, problem solving, goal setting, boundaries), practicing of skills using case studies and feedback from supervisors and other trainees, and resources from | Group members were given a study questionnaire pack, which included outcome measures, an information sheet providing details of the study including its requirements, rationale and potential benefits and risks, and a consent form | Not specified |
| What (procedures) | General education on causes and consequences of stroke was provided as part of a peer support group; patients were invited to share problems experienced in their daily lives | General education on causes and consequences of stroke was provided as part of a peer support group; patients were invited to share problems experienced in their daily lives | Provided targeted stroke-specific information each week and revisited information provided in other weeks to ensure retention of learning and skills | Mentoring sessions with discussions focusing on emotional well-being, post-traumatic brain injury quality of life, and community integration; mentors helped mentees gain access to community resources and discussed TBI- or caregiving-related topics | Peer support sessions during which participants identified topics related to post-stroke rehabilitation that they wanted to focus on; topics identified were common psychosocial difficulties and practical considerations and were often discussed in more than 1 session | Outings fostered increased social networking for the peer partner and involved introductions to people, activities, and resources within the peer partner’s own community; phone calls and emails could be used to supplement required in-person meetings |
| Who provided | Delivered by a psychologist; specific training of the psychologist not specified | Delivered by a psychologist; specific training of the psychologist not specified | Delivered by peer leaders and health professionals (i.e., stroke educators); training provided by the National Stroke Foundation | Delivered by peer mentors; mentors participated in 20 hours of training, including modelling interviewing skills with a supervisor and fellow trainees, telephone role playing, discussion of what is and what is not mentoring, and communication skills and active listening; mentors were evaluated by training staff on social competency, willingness to talk openly about disability and life experiences, motivation, and commitment to participation | Delivered by peer supporters and clinicians; peer supporters attended a 3-hour training session facilitated by the author and study supervisor alongside clinicians in the local service, consisting of information about the proposed group, theoretical knowledge/rationale (about stroke and peer support as a model), and group facilitation skills, involving a combination of teaching, working in pairs, observation, and role-play | Delivered by social peer mentors; social peer mentors received initial training led by a neuropsychologist and a consumer representative with TBI who was part of the research team; training was conducted in 2-hour group sessions including didactic presentation, discussion, and role-play of specific skill-building activities to ensure an understanding of the mentor role, introduce and practice specific content for the facilitation of skill building by the peer partner, review safety issues and how to handle crisis situations, and understand documentation responsibilities |
| How | Delivered face-to-face; provided in a group | Delivered face-to-face; provided in a group | Delivered face-to-face; provided in a group | Delivered by phone calls, emails, or in-person meetings; provided individually | Delivered face-to-face; provided in a group | Delivered face-to-face, by phone call (supplement), and by email (supplement); provided individually |
| Where | Peer support group meetings were held at rehabilitation centres | Peer support group meetings were held at rehabilitation centres | Group sessions were held at a hospital | The intervention was delivered in the community | Peer support groups sessions occurred at a day hospital; the physical environment was chosen to accommodate ambulance transport, wheelchair access, disability parking, a quiet therapeutic space, and a comfortable waiting area to allow for physical, sensory, communication, and cognitive difficulties that group members may have experienced post-stroke | Outings occurred within the peer partner’s own community |
| When and how much | The peer support group met twice per week for a total of 9 sessions, each lasting 1 hour | The peer support group met twice per week for a total of 9 sessions, each lasting 1 hour | The program ran once per week for a total of 8 sessions, each lasting 2.5 hours | The intervention lasted 1 year; contact was made a minimum of once per week for the first month, twice per week for the next 2-3 months, and once per month for the remainder of the year, and had no specified length | The intervention ran once per week for a total of 5 sessions, each lasting for 1.5-2 hours | Outings occurred twice per month for 3 months; the duration of outings was not specified |
| Tailoring | Not specified | Not specified | Not specified | During the intervention, contact more frequent than the minimum guidelines was encouraged; contact was also encouraged beyond the 1-year study period if desired | During the first group session, participants identified topics specific to post-stroke rehabilitation that they wanted to focus on in future sessions, and the identified topics were often discussed in more than one session; group members also had the option of approaching peers and/or professionals at the day hospital after a group session for any further discussions on an individual basis | During the active peer-mentoring phase, an on-call therapist spoke to social peer mentors to discuss the status of the mentor-partner relationship, often assisting with problem-solving and troubleshooting impediments to the match relationship, including problems establishing contact with the peer partner, transportation difficulties, financial concerns, and selection of activities for mentor-partner outings |
| Modifications | Not specified | Not specified | Not specified | Not specified | Not specified | Not specified |
| How well (planned) | The number of patients randomized to the peer support group, but which did not receive the intervention or which were lost to follow-up was assessed | The number of patients randomized to the peer support group but which did not receive the intervention or which were lost to follow-up was assessed | Intervention adherence was assessed by participation; the number of patients randomized to the SSMP but which were not booked to start, did not start, or did not complete the intervention was assessed | The attrition rate was assessed for participants (i.e., individuals with TBI and their significant others) | Peer support group adherence was assessed by participation; the number of participants that provided data were assessed | The number of patients randomized to the peer support group but which did not receive the intervention was assessed |
| How well (actual) | Of the patients randomized to the peer support group (n=76), 69 (91%) received peer support, 4 (5%) did not receive peer support, and 3 (4%) were lost to follow-up | Of the patients randomized to the peer support group (n=76), 67 (88%) received peer support, 4 (5%) did not receive peer support, and 5 (7%) were lost to follow-up | Of the participants randomized to the SSMP (n=48), 25 (52%) completed the SSMP, 2 (4%) did not complete the SSMP, 11 (23%) were not booked to start the SSMP, and 10 (21%) did not start the SSMP | The attrition rate of the participants (i.e., individuals with TBI and their caregivers) in the peer mentoring intervention was 20% | Data was obtained from three separate peer support groups; of the participants allocated to peer support group I (n=12), 6 (50%) provided data, of the participants allocated to peer support group II (n=12), 10 (83%) provided data, and of the participants allocated to peer support group III (n=20; automatically allocated from peer support groups I and II), 7 (35%) provided data | Of the patients randomized to the active peer mentoring group (n=24), 12 (50%) completed mentoring and follow-up, 2 (8%) were excluded due to later-identified medical or behavioural issues, 5 (21%) withdrew at the time of mentor matching, 4 (17%) were unable to be contacted at the time of mentor matching, and 1 (4%) discontinued due to dissatisfaction with mentor pairing |

TBI: traumatic brain injury

*Extension of Aben et al.’s 2013 study to include a 1-year follow-up period.

References

1. Hoffmann TC, Glasziou PP, Boutron I, Milne R, Perera R, Moher D, Altman DG, Barbour V, Macdonald H, Johnston M *et al*: **Better reporting of interventions: template for intervention description and replication (TIDieR) checklist and guide**. *The BMJ* 2014, **348**:g1687.

2. Aben L, Heijenbrok-Kal MH, Loon EMPv, Groet E, Ponds RWHM, Busschbach JJV, Ribbers GM: **Training Memory Self-efficacy in the Chronic Stage After Stroke: A Randomized Controlled Trial**. *Neurorehabilitation and Neural Repair* 2013, **27**(2):110-117.

3. Aben L, Heijenbrok-Kal MH, Ponds RWHM, Busschbach JJV, Ribbers GM: **Long-Lasting Effects of a New Memory Self-efficacy Training for Stroke Patients: A Randomized Controlled Trial**. *Neurorehabilitation and Neural Repair* 2014, **28**(3):199-206.

4. Cadilhac DA, Hoffmann S, Kilkenny M, Lindley R, Lalor E, Osborne RH, Batterbsy M: **A Phase II Multicentered, Single-Blind, Randomized, Controlled Trial of the Stroke Self-Management Program**. *Stroke* 2011, **42**:1673-1679.

5. Hanks RA, Rapport LJ, Wertheimer J, Koviak C: **Randomized Controlled Trial of Peer Mentoring for Individuals With Traumatic Brain Injury and Their Significant Others**. *Archives of Physical Medicine and Rehabilitation* 2012, **93**:1297-1304.

6. Stamatakis CV: **The Efficacy of Peer Support in Community Stroke Rehabilitation**. Cardiff University; 2015.

7. Struchen MA, Davis LC, Bogaards JA, Hudler-Hull T, Clark AN, Mazzei DM, Sander AM, Caroselli JS: **Making Connections After Brain Injury: Development and Evaluation of a Social Peer-Mentoring Program for Persons With Traumatic Brain Injury**. *The Journal of Head Trauma Rehabilitation* 2011, **26**(1):4-19.
