# Supplementary material for: Peer support interventions for individuals with acquired brain injury, cerebral palsy, and spina bifida: a systematic review
Source: BMC Health Serv Res. 2019 May 8;19:288. doi: 10.1186/s12913-019-4110-5 (PMC6505073; doi:10.1186/s12913-019-4110-5)
Supplement: Supplementary file 2 — Table S1. Summary of peer support interventions (n = 6) using the TIDieR framework. [1] (DOC 46 kb) [file 12913_2019_4110_MOESM2_ESM.doc]

Database: Ovid MEDLINE: Epub Ahead of Print, In-Process & Other Non-Indexed Citations, Ovid MEDLINE® Daily and Ovid MEDLINE® <1946-Present>

Search Strategy:

--------------------------------------------------------------------------------

1 Cerebral Palsy/ [ childhood onset disabilities ]

2 (cerebral adj pals$).mp.

3 (little$ adj disease).mp.

4 (hemiplegi$ adj5 spastic$).mp.

5 (dipleg$ adj5 spastic$).mp.

6 (quadriplegi$ adj5 spastic$).mp.

7 (unilateral$ adj5 spastic$).mp.

8 (hemiplegi$ adj3 ataxi$).mp.

9 (dipleg$ adj3 ataxi$).mp.

10 (quadriplegi$ adj3 ataxi$).mp.

11 (unilateral$ adj3 ataxi$).mp.

12 exp Spinal Dysraphism/

13 (spina adj bifida).mp.

14 Meningomyelocele/

15 Meningocele/

16 myelomeningocele.mp.

17 meningocele.mp.

18 lipomyelomeningocele.mp.

19 (spinal adj dysraph$).mp.

20 Brain Injuries/

21 Craniocerebral Trauma/

22 Brain Hemorrhage, traumatic/

23 Brain Injuries, Diffuse/

24 Brain Injuries, Traumatic/

25 Brain Injury, Chronic/

26 Epilepsy, Post-Traumatic/

27 exp Stroke/

28 exp Hypoxia, Brain/

29 Brain Damage, Chronic/

30 Hematoma, Subdural, Intracranial/

31 Hematoma, Epidural, Cranial/

32 exp Meningitis/

33 exp Encephalitis/

34 apoplexy.mp.

35 concus$.mp.

36 encephalitis.mp.

37 meningitis.mp.

38 (cerebrovascular adj accident$).mp.

39 (brain adj2 injur$).mp.

40 (head adj2 injur$).mp.

41 (intracran$ adj2 injur$).mp.

42 (cerebr$ adj2 injur$).mp.

43 (cerebellar adj2 injur$).mp.

44 (brainstem adj2 injur$).mp.

45 (crani$ adj2 injur$).mp.

46 (skull$ adj2 injur$).mp.

47 (hemisphere adj2 injur$).mp.

48 (orbit$ adj2 injur$).mp.

49 (brain adj2 isch?em$).mp.

50 (head adj2 isch?em$).mp.

51 (intracran$ adj2 isch?em$).mp.

52 (cerebr$ adj2 isch?em$).mp.

53 (cerebellar adj2 isch?em$).mp.

54 (brainstem adj2 isch?em$).mp.

55 (crani$ adj2 isch?em$).mp.

56 (skull$ adj2 isch?em$).mp.

57 (hemisphere adj2 isch?em$).mp.

58 (orbit$ adj2 isch?em$).mp.

59 (brain adj2 infarc$).mp.

60 (head adj2 infarc$).mp.

61 (intracran$ adj2 infarc$).mp.

62 (cerebr$ adj2 infarc$).mp.

63 (cerebellar adj2 infarc$).mp.

64 (brainstem adj2 infarc$).mp.

65 (crani$ adj2 infarc$).mp.

66 (skull$ adj2 infarc$).mp.

67 (hemisphere adj2 infarc$).mp.

68 (orbit$ adj2 infarc$).mp.

69 (brain adj2 thrombo$).mp.

70 (head adj2 thrombo$).mp.

71 (intracran$ adj2 thrombo$).mp.

72 (cerebr$ adj2 thrombo$).mp.

73 (cerebellar adj2 thrombo$).mp.

74 (brainstem adj2 thrombo$).mp.

75 (crani$ adj2 thrombo$).mp.

76 (skull$ adj2 thrombo$).mp.

77 (hemisphere adj2 thrombo$).mp.

78 (orbit$ adj2 thrombo$).mp.

79 (brain adj2 emboli$).mp.

80 (head adj2 emboli$).mp.

81 (intracran$ adj2 emboli$).mp.

82 (cerebr$ adj2 emboli$).mp.

83 (cerebellar adj2 emboli$).mp.

84 (brainstem adj2 emboli$).mp.

85 (crani$ adj2 emboli$).mp.

86 (skull$ adj2 emboli$).mp.

87 (hemisphere adj2 emboli$).mp.

88 (orbit$ adj2 emboli$).mp.

89 (brain adj2 h?emorrhag$).mp.

90 (head adj2 h?emorrhag$).mp.

91 (intracran$ adj2 h?emorrhag$).mp.

92 (cerebr$ adj2 h?emorrhag$).mp.

93 (cerebellar adj2 h?emorrhag$).mp.

94 (brainstem adj2 h?emorrhag$).mp.

95 (crani$ adj2 h?emorrhag$).mp.

96 (skull$ adj2 h?emorrhag$).mp.

97 (hemisphere adj2 h?emorrhag$).mp.

98 (orbit$ adj2 h?emorrhag$).mp.

99 (brain adj2 h?ematoma$).mp.

100 (head adj2 h?ematoma$).mp.

101 (intracran$ adj2 h?ematoma$).mp.

102 (cerebr$ adj2 h?ematoma$).mp.

103 (cerebellar adj2 h?ematoma$).mp.

104 (brainstem adj2 h?ematoma$).mp.

105 (crani$ adj2 h?ematoma$).mp.

106 (skull$ adj2 h?ematoma$).mp.

107 (hemisphere adj2 h?ematoma$).mp.

108 (orbit$ adj2 h?ematoma$).mp.

109 (brain adj2 aneurysm$).mp.

110 (head adj2 aneurysm$).mp.

111 (intracran$ adj2 aneurysm$).mp.

112 (cerebr$ adj2 aneurysm$).mp.

113 (cerebellar adj2 aneurysm$).mp.

114 (brainstem adj2 aneurysm$).mp.

115 (crani$ adj2 aneurysm$).mp.

116 (hemisphere adj2 aneurysm$).mp.

117 (orbit$ adj2 aneurysm$).mp.

118 (brain adj2 hypoxi$).mp.

119 (head adj2 hypoxi$).mp.

120 (intracran$ adj2 hypoxi$).mp.

121 (cerebr$ adj2 hypoxi$).mp.

122 (cerebellar adj2 hypoxi$).mp.

123 (brainstem adj2 hypoxi$).mp.

124 (crani$ adj2 hypoxi$).mp.

125 (skull$ adj2 hypoxi$).mp.

126 (hemisphere adj2 hypoxi$).mp.

127 (orbit$ adj2 hypoxi$).mp.

128 (brain adj2 trauma$).mp.

129 (head adj2 trauma$).mp.

130 (intracran$ adj2 trauma$).mp.

131 (cerebr$ adj2 trauma$).mp.

132 (cerebellar adj2 trauma$).mp.

133 (brainstem adj2 trauma$).mp.

134 (crani$ adj2 trauma$).mp.

135 (skull$ adj2 trauma$).mp.

136 (hemisphere adj2 trauma$).mp.

137 (orbit$ adj2 trauma$).mp.

138 (brain adj2 lesion$).mp.

139 (head adj2 lesion$).mp.

140 (intracran$ adj2 lesion$).mp.

141 (cerebr$ adj2 lesion$).mp.

142 (cerebellar adj2 lesion$).mp.

143 (brainstem adj2 lesion$).mp.

144 (crani$ adj2 lesion$).mp.

145 (skull$ adj2 lesion$).mp.

146 (hemisphere adj2 lesion$).mp.

147 (orbit$ adj2 lesion$).mp.

148 (brain adj2 damage$).mp.

149 (head adj2 damage$).mp.

150 (intracran$ adj2 damage$).mp.

151 (cerebr$ adj2 damage$).mp.

152 (cerebellar adj2 damage$).mp.

153 (brainstem adj2 damage$).mp.

154 (crani$ adj2 damage$).mp.

155 (skull$ adj2 damage$).mp.

156 (hemisphere adj2 damage$).mp.

157 (orbit$ adj2 damage$).mp.

158 (brain adj2 oedema$).mp.

159 (head adj2 oedema$).mp.

160 (intracran$ adj2 oedema$).mp.

161 (cerebr$ adj2 oedema$).mp.

162 (cerebellar adj2 oedema$).mp.

163 (brainstem adj2 oedema$).mp.

164 (crani$ adj2 oedema$).mp.

165 (skull$ adj2 oedema$).mp.

166 (hemisphere adj2 oedema$).mp.

167 (orbit$ adj2 oedema$).mp.

168 (brain adj2 edema$).mp.

169 (head adj2 edema$).mp.

170 (intracran$ adj2 edema$).mp.

171 (cerebr$ adj2 edema$).mp.

172 (cerebellar adj2 edema$).mp.

173 (brainstem adj2 edema$).mp.

174 (crani$ adj2 edema$).mp.

175 (skull$ adj2 edema$).mp.

176 (hemisphere adj2 edema$).mp.

177 (orbit$ adj2 edema$).mp.

178 (brain adj2 fracture$).mp.

179 (head adj2 fracture$).mp.

180 (intracran$ adj2 fracture$).mp.

181 (cerebr$ adj2 fracture$).mp.

182 (cerebellar adj2 fracture$).mp.

183 (brainstem adj2 fracture$).mp.

184 (skull$ adj2 fracture$).mp.

185 (brain adj2 contusion$).mp.

186 (head adj2 contusion$).mp.

187 (intracran$ adj2 contusion$).mp.

188 (cerebr$ adj2 contusion$).mp.

189 (cerebellar adj2 contusion$).mp.

190 (brainstem adj2 contusion$).mp.

191 (crani$ adj2 contusion$).mp.

192 (hemisphere adj2 contusion$).mp.

193 (orbit$ adj2 contusion$).mp.

194 (brain adj2 pressur$).mp.

195 (head adj2 pressur$).mp.

196 (intracran$ adj2 pressur$).mp.

197 (cerebr$ adj2 pressur$).mp.

198 (cerebellar adj2 pressur$).mp.

199 (brainstem adj2 pressur$).mp.

200 (crani$ adj2 pressur$).mp.

201 (hemisphere adj2 pressur$).mp.

202 (orbit$ adj2 pressur$).mp.

203 or/1-202

204 Peer Group/ [ Peer Support ]

205 Volunteers/

206 peer.tw.

207 peers.tw.

208 volunteer?.tw.

209 advice.tw.

210 advis$.tw.

211 (health adj coach$).tw.

212 counsel$.tw.

213 (lay$ adj led).tw.

214 (lay$ adj run).tw.

215 (lay$ adj help$).tw.

216 (lay$ adj support$).tw.

217 (lay$ adj visit$).tw.

218 (lay$ adj based).tw.

219 (lay$ adj deliver$).tw.

220 (user$ adj led).tw.

221 (user$ adj run).tw.

222 (user$ adj based).tw.

223 layperson$.tw.

224 (lay adj person$).tw.

225 (community adj person$).tw.

226 (support adj person$).tw.

227 (community adj based).tw.

228 (community adj visit$).tw.

229 (home adj based).tw.

230 (home adj visit$).tw.

231 (expert adj patient?).tw.

232 (non adj professional?).tw.

233 nonprofessional?.tw.

234 (non adj medical).tw.

235 nonmedical.tw.

236 (mutual adj aid).tw.

237 (mutual adj support?).tw.

238 (supportive adj relationship).tw.

239 mentor$.tw.

240 (community adj worker?).tw.

241 (lay adj worker?).tw.

242 (support adj worker?).tw.

243 (voluntary adj work$).tw.

244 (voluntary adj care).tw.

245 (voluntary adj involvement).tw.

246 (voluntary adj help$).tw.

247 (voluntary adj counsel$).tw.

248 or/204-247

249 (clinical adj trial).mp.

250 Clinical Trial.pt.

251 random$.mp.

252 tu.xs.

253 or/249-252

254 203 and 248 and 253

255 exp Animals/ not (exp Animals/ and Humans/)

256 254 not 255

257 limit 256 to english language

258 limit 257 to yr="2008-2018"
